# Single-site, five-year experience with human eosinophil isolation by density gradient centrifugation and CD16 immunomagnetic negative separation

**DOI:** 10.1186/s13104-020-05055-9

**Published:** 2020-04-10

**Authors:** Yun Cao, Sooncheon Shin, Daniela J. Carroll, Jeremy A. O’Sullivan, Bruce S. Bochner

**Affiliations:** 1 Department of Medicine, Division of Allergy and Immunology, Northwestern University Feinberg School of Medicine, Chicago, IL 60611 USA; 2 Division of Allergy and Immunology, Northwestern University Feinberg School of Medicine, 240 E. Huron St., Room M-306, Chicago, IL 60611 USA

**Keywords:** Human eosinophil purification, Blood, Methods, Purity, Yield, Immunomagnetic separation

## Abstract

**Objective:**

Little has been reported regarding the reliability of methods for the purification of human blood eosinophils. We retrospectively reviewed our experience with 350 consecutive eosinophil isolations.

**Results:**

Between January 2014 and December 2018, we conducted 350 eosinophil purifications from 83 donors. Absolute eosinophil count (AEC), calculated from hospital complete blood counts when available (n = 289), ranged from 32 to 1352 eosinophils/µL ($\bar{x} \pm {\text{SD}}$: 179 ± 136/µL). Eosinophil yields ranged from 0.4 to 24.4 million cells per 20 mL of blood drawn ($\bar{x} \pm {\text{SD}}$: 3.1 ± 1.9 million eosinophils) with > 98% purity. Comparing AEC to actual yield, recovery was 87% ± 29% ($\bar{x} \pm {\text{SD}}$) and AEC strongly correlated with yield. To explore the reproducibility of yield, a subsequent analysis was limited to those donors drawn ≥ 3 times (N = 35), and there was no difference in the average coefficient of variation for yield between allergic and non-allergic donors. Viability of isolated eosinophils was consistently > 95% and after 24 h of culture did not differ between allergic and non-allergic donors. We conclude that this immunomagnetic separation method for human eosinophil isolation from whole blood is a reliable, reproducible technique for obtaining an average of 87% yield with high purity and viability.

## Introduction

Human eosinophils, one of the less common types of leukocytes, are felt to contribute to homeostatic, immune and pathologic conditions [1]. Normal numbers of eosinophils in the blood are typically defined as ≤ 500/µL. Blood eosinophilia, defined as an absolute eosinophil count > 500 µL, and hypereosinophilia, defined as any absolute eosinophil count ≥ 1500/µL, can be seen in a variety of conditions ranging from atopic, gastrointestinal and parasitic diseases to drug reactions, immune deficiencies, malignancies and hematopoietic disorders [2, 3]. Because of the potential for eosinophils to directly cause tissue damage, several therapies have been developed that selectively target eosinophils, and as a result, we now know that eosinophils directly contribute to disease pathophysiology in disorders ranging from asthma to eosinophilic granulomatosis with polyangiitis to hypereosinophilic syndromes [4–10].

Our knowledge of eosinophil biology has greatly benefited from the availability of methods that allow their purification for study in vitro. These methods initially involved intricate, multi-step density gradient centrifugation techniques, as eosinophils have the highest density of all leukocytes, overlapping in this regard only with neutrophils [11]. Major advances in the field occurred with the development of activation-based and antibody-based positive and negative selection methods, such as those that incorporate so-called immunomagnetic approaches [12–14], as well as the use of flow cytometric cell sorting [15]. However, little has been reported regarding their reproducibility and yield, as well as donor-to-donor variability and within-donor reproducibility across multiple donors and blood draws. Therefore, we have retrospectively reviewed our experience over a five-year period with 350 consecutive human eosinophil isolation preparations from whole blood using density gradient centrifugation, red blood cell (RBC) hypotonic lysis and immunomagnetic removal of contaminating neutrophils using CD16 antibody and herein describe key features of this methodologic approach.

## Main text

### Methods

#### Human blood donors

Written informed consent for blood donation was obtained using an institutional review board-approved protocol at the Northwestern University Feinberg School of Medicine. The allergic status of donors was determined based on their history (food allergy, atopic dermatitis, allergic rhinitis, and/or asthma) together with a history of needing medications (either actively at the time of the blood draw or on an as-needed basis) for one or more of these conditions. Donors were not allowed to give blood if they had received systemic corticosteroids in the prior 2 months or if they were receiving biologics for any of these conditions within the past year. For most blood donations, a separate vial of blood was drawn and provided to our hospital laboratory so that a complete blood count with differential (CBC) could be obtained. Information from the CBC results were used to determine starting blood eosinophil counts (cells/mm^3^) for calculation of yield, as well as to screen for any hematologic abnormalities such as anemia.

#### Eosinophil isolation

Eosinophils were isolated from mildly allergic and nonallergic donors essentially as described [16]. In brief, 0.1 M EDTA-anticoagulated blood (4 mL per 60 mL of blood) was diluted 1:3 with phosphate-buffered saline, then quantities of 40 mL of diluted blood were layered onto a 10 mL cushion of Percoll (Sigma-Aldrich, St. Louis, MO) adjusted to a specific density of 1.090 gm/mL in 50 mL conical tubes and centrifuged at 335*g* for 20 min at room temperature without braking (Beckman Coulter Allegra model X-15R). Mononuclear cells, platelets and basophils in the upper layer were removed. Granulocytes and RBCs were collected from the pellets, and a 30-s RBC lysis with ice-cold water was performed three times consecutively. Finally, anti-human CD16 magnetic microbeads (Miltenyi Biotec, Auburn, CA) were added to label neutrophils and negatively select eosinophils over a magnetized mesh column. Purity was determined by cytocentrifugation (Shandon Inc., Pittsburgh, PA) and Kwik-Diff (Thermo Fisher Scientific, Waltham, MA) staining. Viability was determined by flow cytometry (Becton Dickenson LSR II) and 4′6-diamidino-2-phenylindole dihydrochloride (DAPI) exclusion (Thermo Scientific). Cells were cultured in RPMI 1640 medium containing 10% fetal calf serum and 0.5% penicillin/streptomycin (5000 units/mL of penicillin and 5000 µg/mL of streptomycin, Thermo Fisher Scientific) with or without 30 ng/mL recombinant human IL-5 (R&D Systems, Minneapolis, MN) at 37° in 5% CO_2_ for 24 h.

### Statistical analyses

Mean ± standard deviation ($\bar{x} \pm {\text{SD}}$), Pearson correlation coefficients (r), coefficients of variation, Student’s t-test and analysis of covariance were calculated using Excel software (Microsoft, Redmond, WA).

## Results

Between January 2014 and December 2018, we conducted 350 eosinophil purifications from 83 donors, yielding 341 successful purifications. Donors included 24 males and 59 females, ranging from 21–60 years of age. Among these 83 donors, 46 were allergic and 37 were non-allergic based on medical and medication history. Absolute eosinophil counts (AECs), calculated from the CBC results when additional blood was drawn for this purpose (n = 289), averaged 179 ± 136 eosinophils/µL ($\bar{x} \pm {\text{SD}}$).

**Fig. 1:**
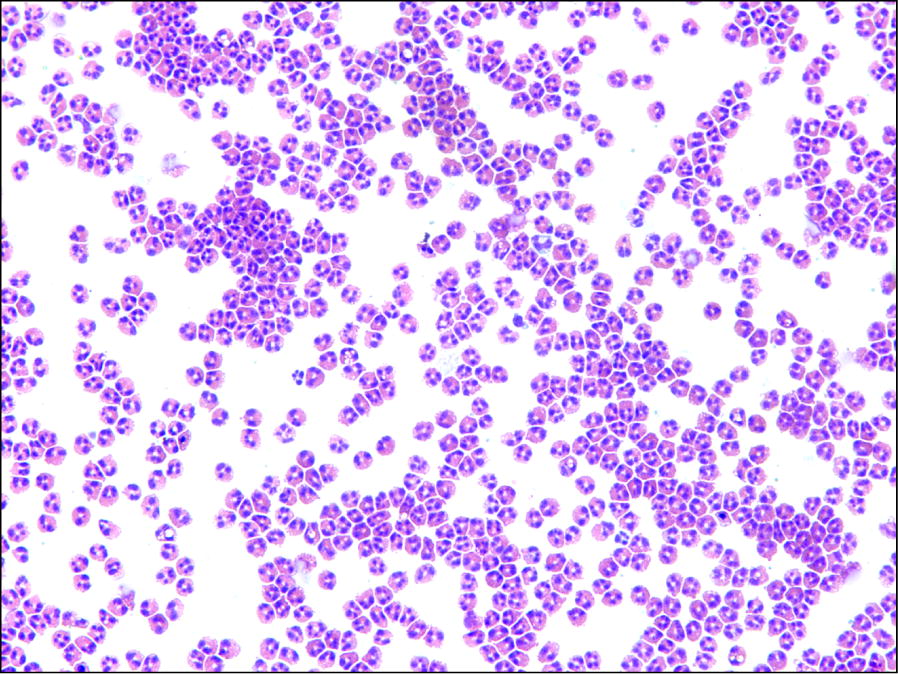
Photomicrograph of a representative Kwik-Diff-stained cytocentrifugation sample of purified eosinophils showing > 98% purity (20× magnification)

**Fig. 2:**
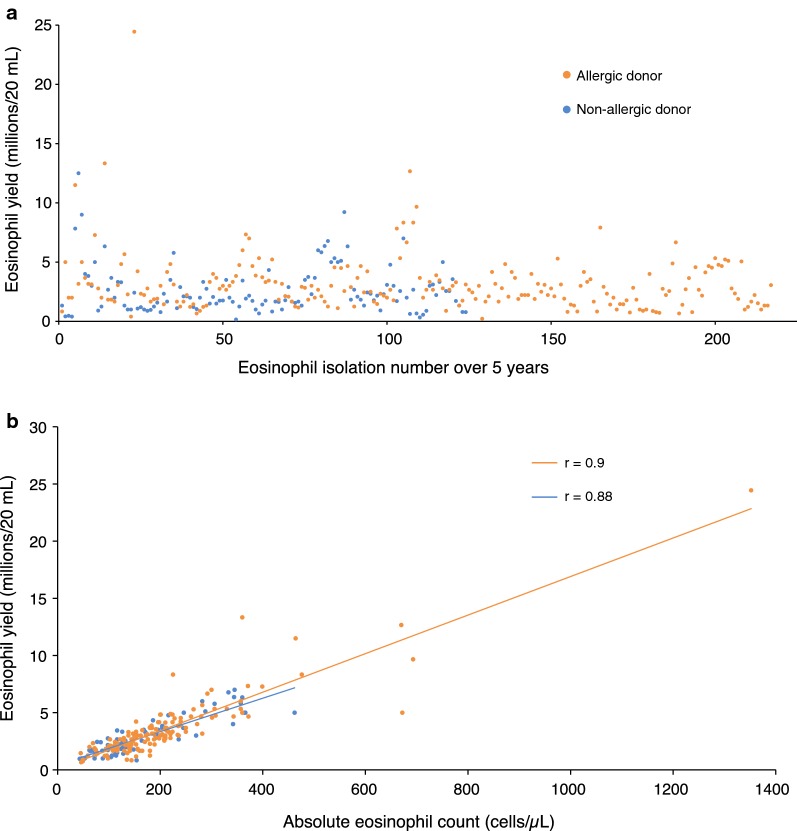
Panel **a**. Eosinophil yield based on allergic status (n = 341 blood draws). Panel **b**. Correlations between eosinophil yield and absolute eosinophil count (n = 289)

**Fig. 3:**
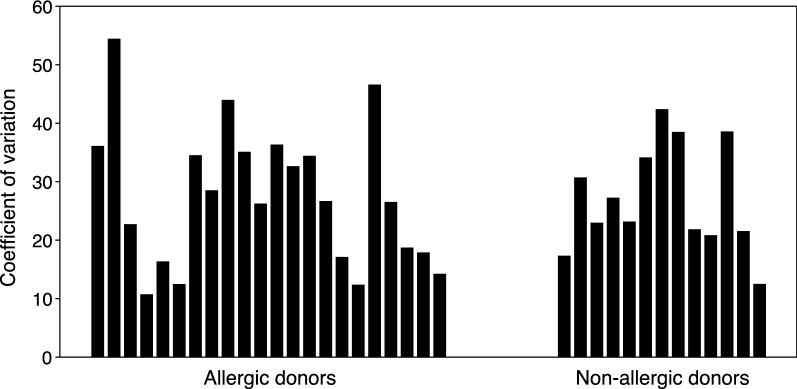
Reproducibility of eosinophil yield for donors drawn ≥ 3 times as assessed by calculations of coefficient of variation (n = 35)

Eosinophil purity following immunomagnetic negative separation was consistently > 98%, with contaminating cells being almost exclusively neutrophils (Fig. 1). Eosinophil yields after 341 out of 350 successful purifications, calculated per 20 mL of blood drawn, ranged from 0.4 to 24.4 million cells ($\bar{x} \pm {\text{SD}}$: 3.1 ± 1.9 million eosinophils, see Fig. 2a). Comparing AEC to actual yield, recovery was 87 ± 29% ($\bar{x} \pm {\text{SD}}$) with a strong linear correlation between AEC and yield that did not differ statistically (p > 0.5 as assessed by analysis of covariance) between allergic and non-allergic donors (Pearson r = 0.9 for allergics, r = 0.88 for non-allergics and r = 0.897 for all 289 donors) (Fig. 2b). To explore the reproducibility of yield, a subsequent analysis was limited to those donors whose blood was drawn ≥ 3 times (N = 35). With this approach, the average coefficient of variation for yield did not differ statistically between allergic (N = 22) and non-allergic (N = 13) donors (27.5% versus 27.1%, respectively, p > 0.5 as assessed by Student’s t-test) (Fig. 3), suggesting that there was no detectable impact of allergy history on eosinophil yield. Initial viability of isolated eosinophils was consistently > 95% and was > 90% after 24 h of culture with 30 ng/mL rhIL-5 versus > 80% without IL-5, and did not differ between allergic and non-allergic donors. Finally, regarding any incidental findings from the CBC results (n = 289), we did note the following: mild lymphopenia (n = 1), neutropenia (n = 4) or thrombocytopenia (n = 2); anemia (n = 4); slight ovalocytes (n = 1), and large platelets (n = 1).

## Discussion

To our knowledge, this report is the largest of its kind describing the results of commonly used methods for human eosinophil isolation from blood. Average eosinophil yield per 20 mL of blood was ≈ 3 million eosinophils with almost 90% recovery and consistently > 95% viability and purity. These were similar between allergic and non-allergic donors, as was their viability with or without IL-5 in culture for 24 h. Yields per donor were fairly reproducible from donation to donation. Therefore, the described method for human eosinophil isolation from whole blood using density gradient centrifugation, RBC hypotonic lysis and immunomagnetic removal of neutrophils is a reliable, reproducible method for obtaining eosinophils at high yield, purity and viability.

## Limitations

The main limitations of this study include the fact that all eosinophil isolations were performed at a single academic site by the same two people in the laboratory, with more than 80% being done by the first author. Other limitations are that the atopic status of each donor was not formally confirmed by traditional allergy testing. Most donors were not taking medications at the time of blood donation, so this should not be a limitation of this study.

## Data Availability

The data supporting this publication is available at ImmPort (https://www.immport.org) under study accession SDY1627.

## Abbreviations

AEC: Absolute eosinophil count
CBC: Complete blood count with differential
DAPI: 4′6-diamidino-2-phenylindole dihydrochloride
RBC: Red blood cell
